# The evolution of lung adenocarcinoma precursors is associated with chromosomal instability and transition from innate to adaptive immune response/evasion

**DOI:** 10.21203/rs.3.rs-4396272/v1

**Published:** 2024-05-15

**Authors:** Xin Hu, Bo Zhu, Natalie Vokes, Junya Fujimoto, Frank R. Rojas Alvarez, Simon Heeke, Andre L. Moreira, Luisa M. Solis, Cara Haymaker, Vamsidhar Velcheti, Daniel H. Sterman, Harvey I. Pass, Chao Cheng, Jack J. Lee, Jianhua Zhang, Zhubo Wei, Jia Wu, Xiuning Le, Edwin Ostrin, Iakovos Toumazis, Don Gibbons, Dan Su, Junya Fukuoka, Mara B. Antonoff, David E. Gerber, Chenyang Li, Humam Kadara, Linghua Wang, Mark Davis, John V. Heymach, Samir Hannash, Ignacio Wistuba, Steven Dubinett, Ludmil Alexandrov, Scott Lippman, Avrum Spira, Andrew P. Futreal, Alexandre Reuben, Jianjun Zhang

**Affiliations:** 1 Department of Genomic Medicine, The University of Texas MD Anderson Cancer Center, Houston, TX, 77030, USA.; 2 Departments of Thoracic/Head and Neck Medical Oncology, The University of Texas MD Anderson Cancer Center, Houston, TX, 77030, USA.; 3 Hiroshima University Hospital, Hiroshima 7348551, Japan; 4 Department of Translational Molecular Pathology, The University of Texas MD Anderson Cancer Center, Houston, TX, 77030, USA.; 5 Department of Pathology, New York University Langone Medical Center, New York, 10012, USA; 6 Department of Medical oncology, New York University, New York, 10012, USA; 7 Department of Pulmonary, New York University, New York, 10012, USA; 8 Department of Cardiothoracic Surgery, New York University Langone Medical Center, New York, 10016, USA.; 9 Department of Medicine, Epidemiology and Population Science, Baylor College of Medicine. Houston, TX, 77030, USA; 10 Department of Biostatistics, The University of Texas MD Anderson Cancer Center, Houston, TX, 77030, USA.; 11 Department of Imaging Physics, The University of Texas MD Anderson Cancer Center, Houston, TX, 77030, USA.; 12 Department of General Internal Medicine, The University of Texas MD Anderson Cancer Center, Houston, TX, 77030, USA.; 13 Department of Health Services Research, The University of Texas MD Anderson Cancer Center, Houston, TX, 77030, USA.; 14 Institute of Cancer and Basic Medicine (IBMC), Chinese Academy of Sciences, Hangzhou, 310022, China.; 15 Department of Pathology, Cancer Hospital of the University of Chinese Academy of Sciences, Zhejiang Cancer Hospital, Hangzhou, 310022, China.; 16 Department of Pathology, Nagasaki University Graduate School of Biomedical Sciences, Nagasaki, 8528523, Japan.; 17 Department of Thoracic & Cardiovasc Surgery, The University of Texas MD Anderson Cancer Center, Houston, TX, 77030, USA.; 18 Harold C. Simmons Comprehensive Cancer Center, UT Southwestern Medical Center, Dallas, TX, 75390, USA; 19 Moores Cancer Center, UC San Diego School of Medicine, San Diego, CA, 92037, USA; 20 Department of Clinical Cancer Prevention, The University of Texas MD Anderson Cancer Center, Houston, TX, 77030, USA.; 21 Departments of Medicine and Pathology, University of California Los Angeles and Greater Los Angeles Healthcare System, Los Angeles, CA, 90095, USA; 22 Pathology & Laboratory Medicine, and Bioinformatics, Boston University, Boston, MA, 02215, USA; 23 Lead contact

**Keywords:** lung adenocarcinoma, pre-cancer, cancer evolution, immune evasion, chromosomal instability, stemness, alveolar differentiation

## Abstract

Studying lung adenocarcinoma (LUAD) early carcinogenesis is challenging, primarily due to the lack of LUAD precursors specimens. We amassed multi-omics data from 213 LUAD and LUAD precursors to identify molecular features underlying LUAD precancer evolution. We observed progressively increasing mutations, chromosomal aberrations, whole genome doubling and genomic instability from precancer to invasive LUAD, indicating aggravating chromosomal instability (CIN). Telomere shortening, a crucial genomic alteration linked to CIN, emerged at precancer stage. Moreover, later-stage lesions demonstrated increasing cancer stemness and decreasing alveolar identity, suggesting epithelial de-differentiation during early LUAD carcinogenesis. The innate immune cells progressively diminished from precancer to invasive LUAD, concomitant with a gradual recruitment of adaptive immune cells (except CD8_+_ and gamma-delta T cells that decreased in later stages) and upregulation of numerous immune checkpoints, suggesting LUAD precancer evolution is associated with a shift from innate to adaptive immune response and immune evasion mediated by various mechanisms.

## INTRODUCTION

Lung cancer remains the leading cause of cancer-related mortality globally, in large part due to frequent diagnosis at late-stage with markedly reduced chances for cure. Early detection through low-dose CT-guided lung cancer screening has demonstrated a significant reduction in lung cancer mortality^1^. Meanwhile, widespread adoption of chest CT scans for screening or management of other medical conditions has resulted in a significant surge in the detection of indeterminate pulmonary nodules (IPNs)^1^. While many IPNs are benign, a subset are precursor lesions that may progress to invasive lung adenocarcinoma (LUAD), the most common subtype of lung cancer^2^. These LUAD precursors include atypical adenomatous hyperplasia (AAH), which may progress into preinvasive adenocarcinoma *in situ* (AIS), minimally invasive adenocarcinoma (MIA)^3, 4, 5, 6^ and finally to fully invasive LUAD (ADC). Whereas many IPNs can be resected with minimal morbidity^7^, such invasive intervention may be medically unnecessary if the lesion was destined to remain benign^8^. In addition, up to 25% of patients may harbor multiple IPNs^9, 10^, which makes surgical resection more challenging. While chemoprevention to halt or slow the progression of these LUAD precursors to invasive LUAD is appealing in principle, clinical trials to date have been disappointing^11, 12, 13, 14, 15, 16, 17, 18, 19^. This may be due to a multitude of factors including a lack of biomarkers for risk prediction and lack of effective therapies for early intervention due to our limited understanding of early lung tumorigenesis.

Understanding the molecular mechanisms of the early stages of lung tumorigenesis is essential to discovering new targets for precise diagnosis, prevention, and therapy. However, studying early carcinogenesis of LUAD is challenging primarily due to the scarcity of adequate clinical specimens of precursor lesions, as surgery is not the standard of care. Over the past decade, we and other groups have made extensive efforts to collect and characterize resected LUAD precursors to depict the molecular evolution and associated immune response during early carcinogenesis of LUAD. A series of studies from our group have revealed that LUAD precursors present a simpler molecular landscape^20, 21, 22, 23^, and more active immunity than invasive LUAD^22, 23, 24^. However, previous studies have been limited by the small sample size. In addition, the transcriptomic features of these LUAD precursors of different histologic stages have not been systematically investigated.

In this study, we sought to capture the evolutionary processes of early LUAD carcinogenesis by performing multi-regional whole exome sequencing (WES), whole genome sequencing (WGS) and RNA-sequencing (RNA-seq) on a large cohort of resected LUAD and LUAD precursors of various histologic stages, with the intent to improve our understanding of the molecular and immune alterations associated with the initiation and progression of LUAD precursors.

## RESULTS

### Aggravating chromosomal instability is associated with accumulation of genetic alterations during the neoplastic evolution from precancer to invasive lung adenocarcinoma.

To investigate the genomic alterations during early LUAD carcinogenesis, we analyzed multi-regional whole exome sequencing (WES) data from 472 samples consisting of 213 LUAD and LUAD precursor lesions of various stages (50 AAH, 46 AIS, 70 MIA, and 47 ADC) that presented radiologically as ground glass opacities (GGO) predominant pulmonary nodules (**Supplementary Data 1**). The median exome sequencing coverage was ~300x. We also performed whole genome sequencing (WGS) in a subset of 26 lesions and their matched normal lung tissue with sufficient DNA at a median coverage of ~45x. There was no significant difference in age, smoking status, or sex between different histologic groups (**Supplementary Data 2**).

**Figure 1.**
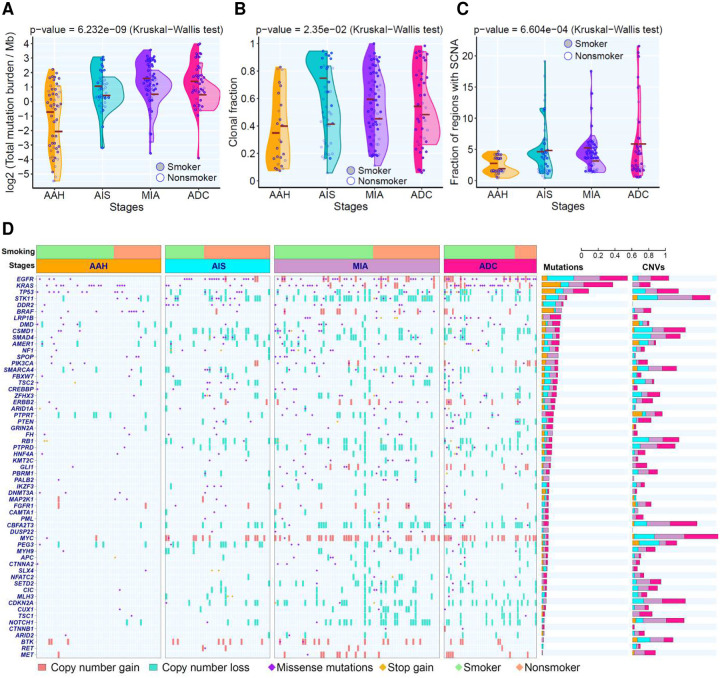
Progressive genomic evolution from AAH to ADC. (**A**) Violin plot of mutational burden across histologic stage. Each point represents the mutational burden in each lesion from smokers (solid) or non-smokers (hollow). Cross bars represent the mean. Kruskal–Wallis H test was used to compare mutational burden across all stages. (**B**) Violin plot showing the proportion of clonal mutations in each lesion. Each point represents the clonal fraction in each lesion from smokers (solid violin) or non-smokers (hollow violin). Cross bars represent the mean clonal fraction. The difference across stages was assessed by Kruskal–Wallis H test. Only lesions with a minimum of 10 SNVs were included for subclonal deconvolution analysis. (**C**) Violin plot of the proportion of chromosomal regions with copy number alterations in each lesion. Each point represents the fraction of chromosomal regions with copy number gain (total copy number > 2.5) or loss (total copy number < 1.5) over the exome capture region across all chromosomes in smokers (solid violin) or non-smokers (hollow violin). Cross bars represent the mean CNV burden. The difference across stages was assessed by Kruskal–Wallis H test. Only regions with a minimum of 50 reads were included to determine copy number alterations. (**D**) The landscape of cancer gene mutations and copy number aberrations in lesions. Cancer gene mutations were defined as nonsynonymous mutations in known cancer genes identical to those hotspots previously reported and stop-gain variants in tumor suppressor genes. Cancer genes located in chromosomal segments with copy number gains (red) or losses (green) are shown. A threshold of focal copy number ≥3 or ≤1 was used to determine chromosomal gains or losses, respectively.

After systematic filtering of single nucleotide variants (SNVs) to remove potential artifacts in formalin-fixed, paraffin-embedded samples, a total of 39,811 mutations from WES were subjected to subsequent analysis. In line with our previous study^20^, we observed higher total mutational burden (TMB) in later-stage lesions (Fig.1A). TMB was higher in smokers compared to non-smokers across all stages (Fig.1A). Subclonal analysis demonstrated that the proportion of clonal mutations was lower in AAH lesions compared to AIS/MIA/ADC. LUAD precursors from smokers exhibited a higher proportion of clonal mutations than non-smokers in all disease stages (Fig.1B). Furthermore, phylogenetic analysis of LUAD precursors with multi-regional WES data revealed a higher proportion of truncal mutations in later-stage lesions (**Fig.S1**). Taken together, these results suggest that the neoplastic transformation of LUAD precancers predominantly occurs as a clonal sweep model^20^.

**Figure 2.**
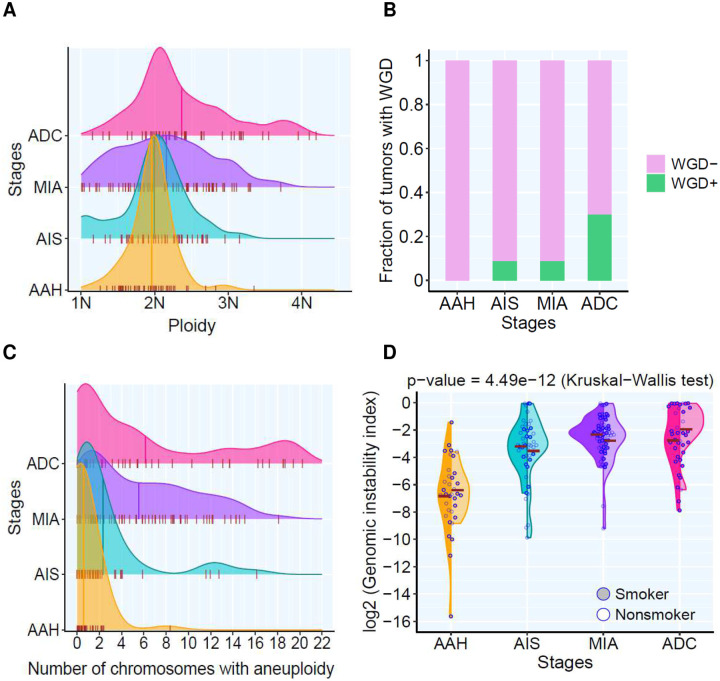
Propagated genomic instability in lesions of different histological stages. (**A**) Density plot showing the distribution of the ploidy among different histological stages. X-axis shows the density of ploidy numbers. The short whiskers show the estimated ploidy value in each lesion. The vertical cross lines show mean ploidy value of all lesions in AAH, AIS, MIA, and ADC, respectively. (**B**) The prevalence of whole genome doubling (WGD) among different histological stages. Each bar represents the proportion of lesions with WGD (green) and without WGD (pink) in each stage. (**C**) Density plot showing the prevalence of the aneuploidy among different histological stages. X-axis shows the number of chromosomes. The short whiskers show the number of chromosomes detected with mosaic aneuploidy in each lesion. The vertical cross lines show mean number of chromosomes carrying aneuploidies from all lesions in AAH, AIS, MIA, and ADC, respectively. (**D**) Weighted genomic instability index (wGII) amongst different histological stages. Each point represents genomic ability index in each lesion, and the brown cross bars represent the mean genomic instability index of all lesions of each histologic stage in smokers (solid) and non-smokers (hollow), respectively. Kruskal-Wallis H test was used to compare genomic instability indexes across stages.

Next, we analyzed somatic copy number alterations (SCNA). SCNA events were observed in AAH frequent lesions, which became more prevalent in AIS, MIA and ADC (Fig.1C, **Fig.S2**). The frequent chromosome arm aneuploidy (CAA) events reported in invasive LUAD^25^, including 1q, 5p and 8q gains and 3p, 8p, 9p, 9q, and 13q losses became prevalent after AIS. Moreover, polyploidy started to emerge at the AIS stage and further expanded in MIA and ADC lesions (Fig.2A). Whole genome doubling (WGD) was not detected in AAH lesions, but it was detected in 9% of AIS lesions, 9% of MIA and 30% of ADC lesions (Fig.2B). In addition, we observed a progressively increased number of chromosomes exhibiting aneuploidy (Fig.2C) and a progressive increase in the weighted genomic instability index (wGII, defined as the fraction of genome altered)^26^ (Fig. 2D) in later-stage lesions. Importantly, wGII was positively correlated with ploidy, SCNA burden and frequency of aneuploidy (**Fig.S3 A–C**) Taken together, these findings suggest accumulated chromosomal instability along with neoplastic progression during early carcinogenesis of LUAD, particularly after malignant transformation post AIS stage.

In addition to SCNA, chromosomal instability can also manifest as structural variants that may have profound impacts on tumor biology^27, 28^. Leveraging WGS data from LUAD precursors of different stages to investigate the timing of structural variants during early LUAD carcinogenesis, we detected structural variants in lesions of all stages (**Fig.S4**), suggesting these may be early genomic events.

The top frequently mutated cancer genes in this cohort of LUAD and precursors included *EGFR*, *KRAS*, *TP53*, *STK11*, and *LRP1B*, most of which emerged at the precancer AAH stage. In addition, copy number losses in tumor suppressor genes (TSG) such as *CDKN2A, TP53, NOTCH1, PTPRD, STK11*, and copy number gain in oncogenes such as *MYC, EGFR*, and *MET* were detected in precancers of all stages but with higher incidence in later-stages (Fig.1D). These observations indicate that worsening chromosomal instability may have led to copy number gain or loss of critical cancer genes, which subsequently may have contributed to the initiation and progression of LUAD precancers.

### Telomere shortening may represent a pivotal early genomic event underlying chromosomal instability during early carcinogenesis of lung adenocarcinoma.

**Figure 3.**
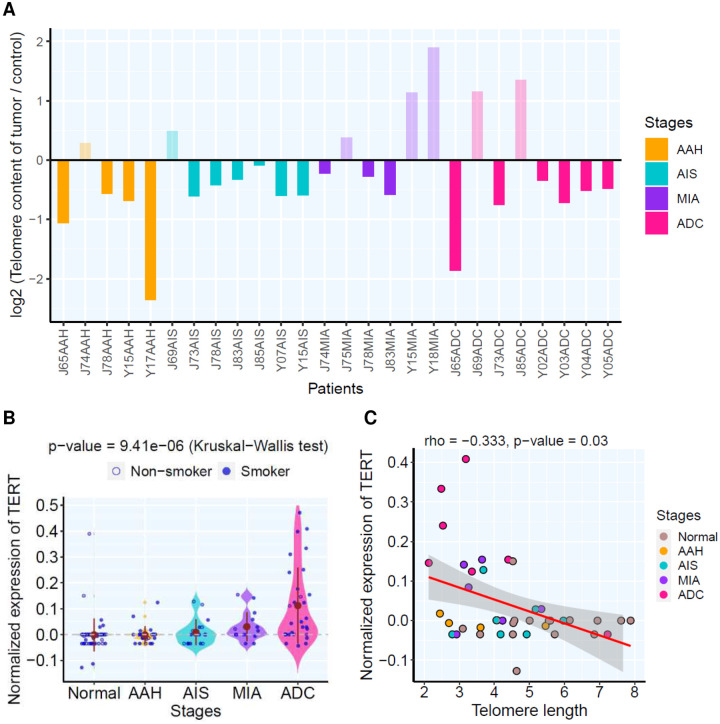
The telomere length and *TERT* expression in each lesion. **(A)** Each bar represents the relative telomere length (RTL) in each lesion based on WGS profiling. **(B)** *TERT* expression amongst different histological stages. Each blue dot represents normalized expression of *TERT* in each pulmonary nodule and the solid brown dots represent the mean expression of all lesions of each histologic stage. Kruskal-Wallis H test was used for comparing normalized *TERT* expression between all stages. **(C)** The correlation of absolute telomere length by WGS profiling and normalized *TERT* expression between lesions (only lesions profiled by both WGS and RNAseq) assessed by two-tailed Spearman’s correlation analysis. Each dot represents each lesion from Normal (brown), AAH (orange), AIS (cyan), MIA (purple) and ADC (rose), respectively.

Telomere length shortening has been reported to be causative of chromosomal insstability^29 30^. In the LUAD precursors for which WGS data was available, telomere shortening (compared to matched normal lung tissues) was a common and early genomic event, which was observed in 19 out of 26 lesions with WGS data available (Fig.3A), including 4 of 5 AAH lesions. In parallel, the expression of telomerase (*TERT*) gradually increased in later-stage lesions (Fig.3B) and negatively correlated with telomere length (Fig.3C). These data indicate that chromosomal instability, an important cancer hallmark, may be an early genomic event during LUAD carcinogenesis that emerges at precancer stage, while telomere shortening is a potential genomic alteration underlying chromosomal instability.

### Evolution from pre-cancer to invasive LUAD is associated with increased transcriptomic intratumor heterogeneity and epithelial dedifferentiation.

**Figure 4.**
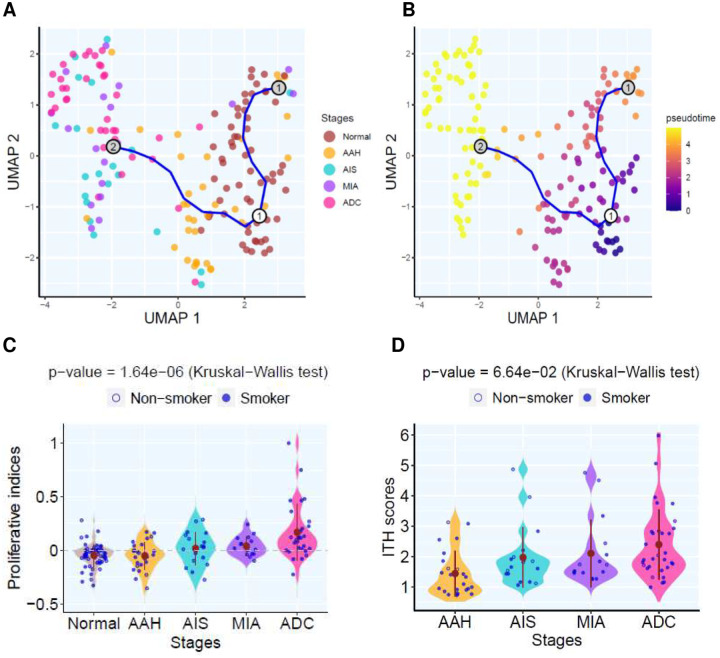
Transcriptomic trajectory and intra-tumoral heterogeneity (ITH) of lesions across distinct pathological stages. (**A**) Pseudotime trajectory was estimated using selected genes with high variance and expression in specimens of different pathological stages. Point colors represent histological stage. (**B**) Trajectory plot colored by estimated pseudo time. (**C**) The proliferative scores amongst different histological stages. Each blue point represents the proliferative indices in each pulmonary nodule and the solid brown points represent the mean proliferative indices of each histologic stage. Kruskal-Wallis H test was used to compare proliferative indices between all stages. (**D**) Transcriptomic ITH scores among different histological stages. Each blue point represents ITH in each pulmonary nodule and the solid brown points represent the mean ITH of each histologic stage. Kruskal-Wallis H test was used to compare ITH scores between all stages.

To understand early LUAD carcinogenesis at the transcriptomic level, we performed RNA sequencing (RNA-Seq) on a subset of 168 LUAD and LUAD precursors with adequate tissue available. Principal component analysis (PCA) and hierarchical clustering displayed distinct clusters between normal lung, AAH and AIS/MIA/ADC (**Fig.S5A–B**) highlighting the transcriptomic divergence at the transition of malignant transformation. Pseudo-time analysis further revealed the evolutionary trajectory from normal lung tissue to AAH, then AIS/MIA/ADC (Fig.4A–B). In addition, the proliferative index based on a pan-cancer proliferative gene signature^31^ (**Supplementary Data 3**) was significantly higher in later-stage than early stage lesions (Fig.4C) indicating increasing proliferation rate along with neoplastic progression. Furthermore, network entropy analysis^32^ to infer transcriptomic intra-tumoral heterogeneity (ITH) uncovered a higher level of transcriptomic ITH in later stage lesions than their early stage counterparts (Fig.4D and **Fig.S6**), in line with the higher degree of heterogeneity in methylation in later stage LUAD precursors^21, 33^.

**Figure 5.**
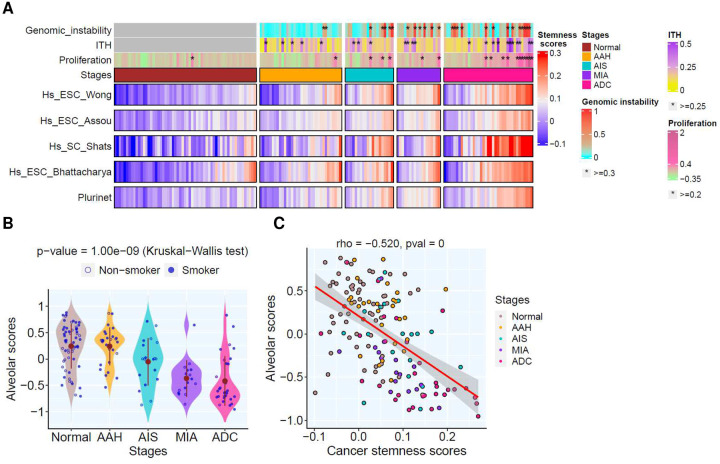
Cancer stemness signatures in lesions of different stages and associated genomic features. (**A**) The top bars show genomic instability scores calculated based on WES allelic copy number data, ITH scores estimated using transcriptomic network entropy; and proliferative scores inferred by gene signature. The stars (*) indicate lesions with genomic features greater than the cut-off values. The heatmap panel shows cancer stemness signatures (derived from different resources) grouped in different stages including AAH (typical adenomatous hyperplasia), AIS (adenocarcinoma in situ), MIA (minimally invasive adenocarcinoma), and ADC (invasive adenocarcinoma). (Source data is provided as a source data file). (**B**) The alveolar scores among different histological stages. Each blue dot represents alveolar score in each pulmonary nodule and the solid brown dots represent the mean alveolar scores within each histologic stage. Kruskal-Wallis H test was used to compare alveolar scores between all stages. (**C**) Correlation of cancer stemness signatures and alveolar scores amongst different histological stages.

One hallmark of cell plasticity in cancers is dedifferentiation, a process whereby tumor cells lose their specialized properties and revert to less differentiated phenotypes reminiscent of early embryonic development or regenerative processes^34^. To understand the dedifferentiation process during early LUAD carcinogenesis, we estimated cancer stem cell (CSC) scores^35^ (**Supplementary Data 3**), which revealed significantly higher CSC scores and pluripotency signaling in later stage lesions (Fig.5A and **Fig.S7A–B**). In parallel, we observed a progressive decrease in the alveolar scores^36^ (Fig.5B, **Supplementary Data 3**). Importantly, the alveolar scores were negatively associated with the CSC scores (Fig.5C). Further analysis revealed that the expression of pluripotency transcription factors such as *FOXM1, OCT4, SOX9, TWIST1*, gradually increased, while *KLF4* gradually decreased in later-stage lesions (**Fig.S8A–E**). These results indicate the dedifferentiation of epithelial cells may be regulated by the core pluripotency stem cell transcriptional factors during early LUAD carcinogenesis.

### Neoplastic progression of LUAD precursors is associated with transition from innate to adaptive immune response and immune evasion.

**Figure 6.**
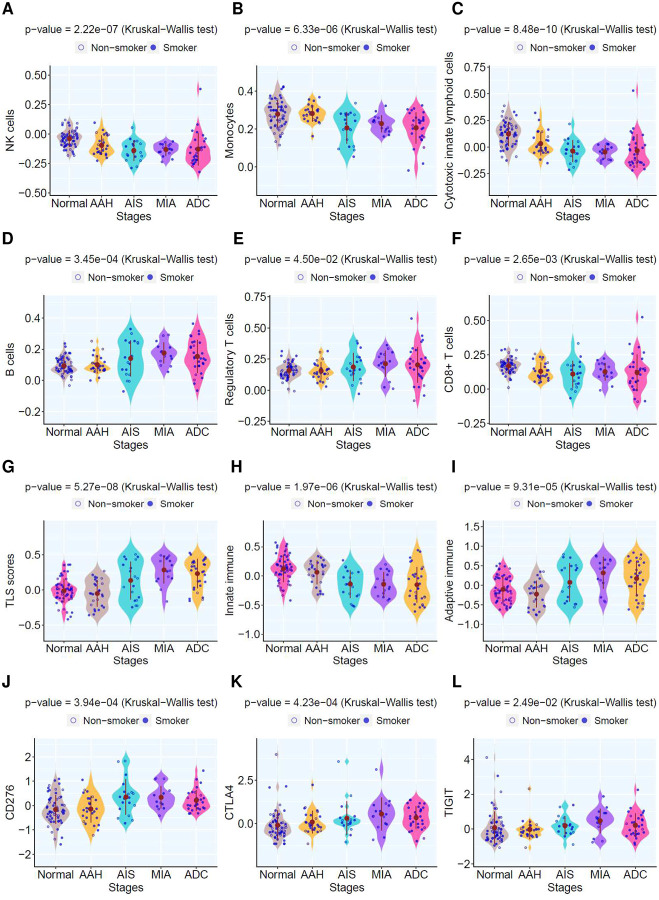
Immune cell infiltration and immune gene expression in lesions of different histological stages. Representative innate immune cell infiltration (**A–C**) and adaptive immune cell infiltration (**D–F**) based on deconvolution using Consensus amongst different histological stages. Each blue dot represents averaged enrichment score inferred in each pulmonary lesion and the solid brown dots represent the mean enrichment score of all lesions in each histologic stage. Kruskal–Wallis H test was used to compare the enrichment score across stages. The GSVA enrichment score of genes associated with tertiary lymphoid structures (TLS) (**G**), innate immunity (**H**) and adaptive immunity (**I**), respectively. (**J–L**) Normalized expression of representative immune checkpoint genes across stage.

The initiation and development of LUAD precancers is influenced by the intricate interplay between evolving neoplastic cells and host factors, particularly anti-tumor immunity^37^. We next leveraged the gene expression data to delve into the immune features of LUAD precursors at various stages. Transcriptomic deconvolution demonstrated reduced infiltration of innate immune cells including NK cells, neutrophils, monocytes, eosinophils, and mast cells in later stage lesions (Fig.6A–B, **Fig.S9A–D**). Conversely, there was an increase in activated myeloid dendritic cells (mDCs) that are known to play important roles in antigen presentation and T cell priming^38^, as well as various adaptive immune cells, such as B cells, plasma cells, regulatory T cells (Treg), with notable exception of CD8_+_ T cells and gamma-delta (γδ) T cells with reduced infiltration in later-stage lesions (Fig.6D–F. **Fig.S9E–H**). The cytotoxic score also decreased in later stages (Fig. 6C).

We further applied GSVA to determine the expression of essential immune genes in LUAD precursors of different stages. Intriguingly, the tertiary lymphoid structure (TLS) score was higher in later-stage lesions (Fig.6G). Correspondingly, the densities of lymphoid follicles, lymphoid aggregates, and TLS, based on pathological assessment, were also higher in later-stage lesions (**Fig.S10, S11**), aligning with previous pathomics analysis^39^. This suggests an organized host anti-tumor immune response amid neoplastic progression of LUAD precursors. Consistent with deconvolution analysis, GSVA revealed a decrease in the expression of innate immunity markers (Fig.6H) and an increase in adaptive immunity in later-stage lesions (Fig.6I). Lastly, numerous immune checkpoints were upregulated in later-stage lesions (Fig.6J–L).

Taken together, these findings imply a transition from innate to adaptive immune response during the neoplastic progression from precancer to frankly invasive LUAD. However, neoplastic cells eventually evade host anti-tumor immunity through multiple mechanisms, including an increase in negative immune regulators such as Tregs and immune checkpoints, as well as downregulation of immune effectors such as cytotoxic lymphocytes, leading to progression into invasive LUAD.

## DISCUSSION

Despite major advances in its treatment, lung cancer remains the leading cause of cancer-related death. There is an urgent need for effective strategies to prevent the development of this deadly malignancy. While risk avoidance, such as smoking cessation, represents a key strategy for reducing lung cancer risk, up to 20% of lung cancer patients are non-smokers^40, 41^. Moreover, among smokers with lung cancer, the majority have quit smoking well before their diagnosis, further highlighting the critical need for alternative active approaches for lung cancer interception^42,43^.

Although it has long been known that LUAD precursors often present as GGO-predominant lung nodules, interception of LUAD has been hindered by our rudimentary understanding of the underlying molecular events and associated tumor microenvironment changes fueling malignant transformation and neoplastic evolution. Recent studies have characterized the molecular and immune features of early-stage LUAD and its precursors^20,21,24,44, 45, 46^. However, the evolutionary trajectory and intricate crosstalk with host immunity during the initiation and progression of LUAD precursors remain understudied. Leveraging a large cohort of resected LUAD precursors through international collaborations, this study aimed to identify the sequential molecular changes driving LUAD precancer initiation and progression, along with the associated immune response and evasion.

We found that critical driver alterations, including canonical *EGFR* and *KRAS* mutations, were detected across the spectrum of lung cancer precursors. This observation underscores the potential for interception of precancerous lesions of LUAD by targeting these early events.

However, a substantial proportion of LUAD precursors lacked targetable driver mutations, presenting a challenge for interception using targeted therapy agents. Alternatively, an immune-based strategy may be more widely applicable, as immune evasion is a universal phenomenon in cancers. Immune prevention has shown success in cancers associated with infectious agents such as hepatitis B^47^ and human papillomavirus^48^. However, applying lung cancer immune prevention faces challenges due to our limited understanding of the evolving interplay between premalignant/malignant cells and the host’s anti-tumor immunity during the formation and progression of pre-cancerous lesions.

Host immunity continuously evolves during cancer development. Our study revealed a dynamic immune response marked by a transition from innate to adaptive immunity with neoplastic progression. During early LUAD carcinogenesis, the broad, non-specific, and rapidly acting innate response serve as the first line of defense. As cancer evolves, anti-tumor immunity gradually transitions to a more specific and potent adaptive immune response both in quantity (higher level of various adaptive immune-cell infiltration) and quality (more organized TLS) in later-stage lesions. Such a transition has also been observed in precancer evolution in oral squamous cell carcinoma^49^ and colorectal cancers^50^. This transition marks the host’s attempt to sustain anti-cancer immune surveillance. However, cancer cells eventually evade immune attacks through mechanisms that include increasing negative regulators (e.g., Tregs and immune checkpoints) and decreasing effectors (e.g., CD8_+_ T cells)

Our findings support a potential role for immune interception of LUAD precursors to prevent lung cancer development. In keeping with this hypothesis, our group has launched two investigator-initiated immune interception trials: Can-Prevent-Lung (NCT04789681, testing reprogramming primarily of innate immunity through canakinumab --anti-IL1β monoclonal antibody treatment) and IMPRINT-Lung (reprogramming adaptive immunity by the anti-PD1 agent pembrolizumab). The planned interim analysis of the Can-Prevent-Lung trial demonstrated that canakinumab has a good safety profile and promising activity in treating persistent high-risk lung nodules^51^. These promising early successes mark a crucial step toward immune interception for lung cancer prevention. The results in the current study suggest that while both innate and adaptive immunity exhibit potential for immune interception, targeting innate immunity may be more efficient at earlier stages, whereas targeting adaptive immunity may have advantages in later-stage lesions. One major challenge is to distinguish early versus late-stage lesions without surgical resection and pathological assessment. Advanced technologies, including liquid biopsies and radiomics approaches^52^, may have the potential to characterize the stage and molecular subtype of lesions for precise immune interception.

Neoplastic progression involves the outgrowth of tumor subclones with reduced immunogenicity, allowing escape from immunosurveillance. In the current study, we observed de-differentiation of epithelial cells during precancerous progression, increased cancer cell stemness, and diminished alveolar epithelial cell identity in later-stage lesions. This phenomenon is consistent with observations in genetically engineered mouse models of LUAD^53^ and other cancers including glioblastoma^54^, intestinal tumors^54^, melanoma^55^, and breast cancer^56^. In principle, immunity has evolved to protect stem cells, which are essential for normal development and tissue homeostasis^57^. Emerging evidence highlights the pivotal role of stemness in immune editing and the evolution of cancer^57^. In the context of LUAD development, increasing stemness may act as a mechanism driving immune evasion, facilitating the transition of LUAD precursors into invasive tumors. Whereas the clinical translation of cancer stem-cell biology is still in its infancy, targeting pre-cancer stem cells may represent a potential cancer interception strategy^57^.

The scarcity of resected LUAD precursor specimens has impeded our understanding of early carcinogenesis of LUAD. This limitation has become a bottleneck hindering the trials aimed at preventing progression to invasive LUAD. Our multi-omics study on a large cohort of resected LUAD unveiled a transition from innate to adaptive immune response during the early neoplastic evolution. These findings have provided biologic support for our ongoing immunoprevention trials targeting innate immunity (Can-Prevent-Lung) and adaptive immunity (IMPRINT-Lung) for lung cancer interception. Future studies are warranted to delve into cell-cell interactions, key cytokines, chemokines, and their gradients at distinct stages of early LUAD carcinogenesis and provide novel insights for the development of novel and effective precision interception strategies.

### Limitations of the study

One important caveat of the current study, common to most other similar studies, is that all the analyses were based on resected specimens, which only provide single molecular snapshots of the evolutionary process of LUAD. While a linear model of evolution from AAH to AIS, MIA, then to ADC was assumed, whether all AAH lesions progress to AIS, MIA, or ADC, and whether every ADC follows the hypothetical linear evolutionary trajectory are unknown. Understanding how the genomic landscape evolves over time with neoplastic progression and its association with patient outcomes requires longitudinal biopsies throughout the disease course, which is impractical in clinical practice. Future studies using animal models or leveraging longitudinal biopsy specimens from interception trials, such as IMPRINT-Lung (NCT03634241) and Can-Prevent-Lung (NCT04789681), may present good opportunities offer to investigate the temporal changes in molecular features during the neoplastic progression of LUAD.

## METHODS

### Patient cohort

A total of 473 resected tumor specimens and 111 matched adjacent normal lung tissue samples were obtained from 111 patients presenting with GGO-predominant lesions by LDCT-guided screening or incidental findings, who underwent surgery at New York University, Nagasaki Hospital (Japan) and Zhejiang Cancer Hospital (China) from 2014 to 2019. None of these patients received preoperative chemotherapy or radiotherapy (**Supplementary Data 1**). 472 specimens were subjected to multi-regional whole exome sequencing. Whole RNA sequencing analysis included a subset of 168 samples and whole genome sequencing included 42 of those specimens, respectively. Available demographic information included patient age at date of specimen collection, age at diagnosis, gender, stated race and ethnicity, smoking status and tumor histology based on two independent pathologists’ review. Written informed consent was obtained from all patients. The analysis was performed using de-identified data under the Institutional Review Boards (IRB) at MD Anderson Cancer Center, New York University, Zhejiang Cancer Hospital and Nagasaki University Graduate School of Biomedical Sciences.

### Next-Generation Sequencing

Manual macro-dissection on the H&E slides of FFPE specimens was performed to ensure a minimum of 40% diseased (atypical or malignant) cells in each multi-region sample based on the region of interest (ROI) diagnosed by the pathologists. Samples with lower disease content were excluded from further analyses. Adjacent normal lung tissue (≥2 cm from tumor margin, morphologically negative for malignant cells) from the same patients was used as germ line control. DNA and RNA were extracted using Ionic^®^ purification system, respectively (Purigen Biosystems). The resulting genomic DNA was processed using Twist NGS Library Preparation and Capture Kits (#104175) with the Human Twist Comprehensive Exome Panel and subjected to whole-exome sequencing (WES) on the S4 flow cell of NovaSeq 6000 system (Illumina) running NovaSeq Control Software v1.7.5 at 150nt paired-end with dual 10 index reads. The whole-genome sequencing (WGS) run at 150nt paired-end was performed on NovaSeq6000 sequencer (Illumina) by Novogene. The RNA library was prepared with TruSeq^®^ Stranded Total RNA Library Prep Gold (#20020599) and subjected to one lane of S4 flow cell on NovaSeq 6000 running NovaSeq Control Software v1.7.5 at 101nt paired end with dual 8 index reads. The demultiplex of both runs was performed using bcl2fastq v2.20.0.

### Single-Nucleotide Variants (SNVs) detection from WES and WGS

Sequencing reads were quality controlled and trimmed by fastp (v0.23.0)^58^, then mapped to the human reference sequence GRCh38 (hg38) using the Burrows-Wheeler Aligner (BWA)-MEM algorithm (v0.7.17). Duplicate reads were marked using Picard (v1.67) followed by realignment around known indels and base quality recalibration was performed using GATK 3.7. Somatic mutation calls were performed using Mutect (v1.1.7), Varscan2 (v2.4.2), Strelka2 (v2.9.2), Lancet (v1.1.0), SomaticSniper (v0.7.4), allowing at least 0.02 variant allele frequency and coverage of ≥ 20 in tumor and up to maximum of 0.01 allele frequency and coverage of ≥ 10 in normal samples. First, we manually curated a trustworthy list of mutations by combining WUST cancer mutations and TCGA_LUAD mutation profiles, to which SNVs matched are preserved from further filtering. Then those variants detected by at least two above callers were selected, and suspicious artifacts due to sequencing errors in FFPE samples were marked by MicroSEC^59^ and SOBDetector^60^. Finally, only single-nucleotide variants (SNVs) 1) detected by at least two callers and 2) not marked as suspicious artifacts and 3) excluded from dbSNP146 and 4) tumor allele frequency >=0.04 and LOD >=10 or included in cosmic database containing census genes were selected. And then the resulting list of somatic SNVs were annotated by multiple databases using Ensembl Variant Effect Predictor (VEP).

### Estimation of telomere length

Telomerehunter (v1.1.0)^61^ was applied to quantify telomere content and composition using 10 telomere variant repeats including TCAGGG, TGAGGG, TTGGGG, TTCGGG, TTTGGG, ATAGGG, CATGGG, CTAGGG, GTAGGG and TAAGGG in matched tumor and normal samples.

### Identification of chromosomal instability related events

Tumor purity was inferred using TITAN framework^62^ and ASCAT^63^, somatic copy number alterations (SCNAs) were detected using CNVkit^64^. The allelic copy number profiles and corresponding ploidy of tumor samples were generated applying “FACETS” packages^65^ using the matched germline data. Whole-Genome Doubling (WGD) was determined (p-value <0.001 for samples with ploidy ≤3) based on random simulation test of WGD. Each sample, s was represented as an aberration profile of major and minor allele copy numbers at chromosome arm resolution. From which *Ns*, the total number of aberrations (relative to diploid) and *Ps*, the probabilities of loss/gain for major and minor allele at each chromosome arm were calculated. 10,000 simulations were run for each sample. In each simulation, *Ns* sequential aberrations, based on *Ps*, were applied to a diploid profile. A *P*-value for genome doubling was obtained by counting the percentage of simulations in which the proportion of chromosome arms with a major allele copy number ≥2 was higher than that observed in the sample. The weighted Genome Instability Index (wGII) was calculated to estimate the proportion of the genome with aberrant copy number compared with the median ploidy, weighted on per-chromosome length basis. The mosaic chromosomal aneuploidies were identified using MAD-seq^66^, based on fitting a mixture model of alternate allele frequencies (AAFs) at heterozygous loci. The subclonal architecture reconstruction was inferred by CliP using a penalized likelihood model^67^.

### Detection of structural variants

Five variant callers were used to identify somatically acquired structural variants from matched tumor and germline whole genome sequencing data: DELLY^68^, LUMPY^69^, BRASS (BReakpoint AnalySiS) (https://github.com/cancerit/BRASS/), Manta^70^ and SVABA^71^. These were merged into a final call set using SURVIVOR^72^, a graph-based algorithm to identify overlapping breakpoint junctions across different callers, accepting all structural-variant calls made by two or more of the five algorithms to obtain best trade-off between sensitivity and specificity. “gGnome” package was used to graph the genomic intervals.

### Bulk RNAseq processing and gene expression matrix construction

Initially, raw sequencing data underwent quality control and adapter trimming using FASTP (v0.20.0). Subsequently, ribosomal RNAs (rRNAs) were eliminated using SortMeRNA^73^, followed by mapping to human transcriptome reference (hg38) using STAR aligner. The expected transcript counts were quantified using RSEM (v1.3.3). Then outliner samples were removed via voom (voomWithQualityWeights) and RSEM transcripts were filtered with a minimum of two counts in all samples and variance stabilized transform (VST, DESeq2) was applied. Batch effects were assessed with Principal Component Analysis (PCA) and removed with LIMMA via linear modeling (removeBatchEffect) using DESeq2 (v1.38.3). The normalization of gene expression matrix was performed by subtracting the median of each transcript across all samples, and only transcripts mapped to coding genes (GENCODE -human release 38) were selected for downstream analysis.

### Pseudotime analysis of specimens diagnosed with different histological stages

The trajectory paths were inferred using “tradeSeq”^74^ and “monocle” packages^74^ on selected genes with high variance and expression across all the samples diagnosed with different pathological stages.

### Tumor heterogeneity analysis using transcriptomic profiling

The transcriptome-based ITH was estimated using nJSD, an entropy-based distance metric between two networks of tumor and matched normal samples, with Jensen-Shannon Divergence (JSD)^32^.

### Deconvolution of tumor infiltrating immune cells

The content of tumor infiltrating NK cells, monocytes, cytotoxicity innate lymphoid cells, neutrophils, eosinophils, activated dendritic cells, B cells, regulatory T cells, CD8_+_ T cells were estimated using Consensus^75^. The mast cells resting, plasma cells were calculated using Cibersort^76^. The infiltration of naïve CD4_+_ T cells, memory CD4_+_ T cells and activated myeloid dendritic cells were inferred using xCell^77^ based on bulk RNAseq expression matrix.

### Gene Set Variation Analysis (GSVA)

The normalized gene expression matrix was then processed to produce ssGSEA enrichment scores by GSVA, which calculates per sample overexpression level of a particular gene list by comparing the ranks of the genes in that list with those of all other genes^78^. A list of gene sets that are functionally associated with proliferation, cancer cell stemness, alveolar differentiation, tertiary lymphoid structures (TLS), innate immunity and adaptive immunity were used as gene signatures (**Supplementary Data 3**). Differential expression at the gene set level was assessed using a multivariate linear model and the empirical Bayes method in LIMMA.

### Assessment of lymph nodes aggregates (LA) and tertiary lymphoid structures (TLS) in H&E-stained image

The archived Hematoxylin and Eosin (H&E) stained pathology slides were first scanned at 20X magnification using Aperio AT2 scanner and uploaded to the digital image analysis software HALO-AI-v3.5 (Indica Labs) (https://indicalab.com/halo-ai/). Then the deep learning tissue classification algorithm was applied to annotate some representative ROIs under the pathologist’s supervision, and the entire tissue section was classified into LA/TLS versus lung tissue, finally LA/TLS were manually assessed for tissue classification accuracy and the numbers of lymph nodes aggregates including TLSs on individual slide were quantified.

### Statistical analysis

All statistical analyses were performed using R software version 4.1.0. Violin plots were generated using “geom_violin” function in ggplot2 (v.0.9.1) to represent data point density along the Y-axis, and the “stat_summary” function from ggplot2 (v.0.9.1) was used to calculate the mean as the center point. Differences in TMB, fraction of clonal mutations, relative telomere length, genomic instability, proliferation, cancer cell stemness and normalized gene expression, immune cell infiltration, TLS scores between the lesions of different stages were assessed using the Kruskal–Wallis H test. Two-sided Spearman’s correlation coefficient was used to access the association between two variables. Confidence intervals for proportions were computed using a 2-sample z-test without continuity correction. All tests were carried out at the 5% significance level with Benjamini-Hochberg correction for multiple testing.

## Data Availability

The raw sequence data has been deposited at European Genome-phenome Archive (EGA), which is hosted by The European Bioinformatics Institute (EBI) and the Centre for Genomic Regulation (CRG) under the accession code: EGAD50000000395 (RNAseq), EGAD50000000396 (WGS), EGAD50000000397 (WES), EGAD00001004960 (WES). Further information about EGA is available at https://ega-archive.org. All other data may be found within the main manuscript or Supplementary Information or available from the authors upon request.
